# Extracellular Vesicles Derived From Colorectal Cancer Affects CD8 T Cells: An Analysis Based on Body Mass Index

**DOI:** 10.3389/fcell.2020.564648

**Published:** 2020-11-26

**Authors:** Nadiah Abu, Norahayu Othman, Nur’ Syahada Ab Razak, Nurul Ainaa’ Adilah Rus Bakarurraini, Siti Nurmi Nasir, Joanne Ern Chi Soh, Luqman Mazlan, Zairul Azwan Mohd Azman, Rahman Jamal

**Affiliations:** ^1^UKM Medical Molecular Biology Institute, UKM Medical Center, Universiti Kebangsaan Malaysia, Kuala Lumpur, Malaysia; ^2^Department of Surgery, UKM Medical Center, Universiti Kebangsaan Malaysia, Kuala Lumpur, Malaysia

**Keywords:** obesity, lymphocytes, microvesicles, anti-tumor immunity, long non-coding RNA

## Abstract

Colorectal cancer (CRC) is one of the most widely diagnosed cancers worldwide. It has been shown that the body-mass index (BMI) of the patients could influence the tumor microenvironment, treatment response, and overall survival rates. Nevertheless, the mechanism on how BMI affects the tumorigenesis process, particularly the tumor microenvironment is still elusive. Herein, we postulate that extracellular vesicles (EVs) from CRC patients and non-CRC volunteers with different BMI could affect immune cells differently, in CD8 T cells particularly. We isolated the EVs from the archived serum of CRC patients with high and low BMI, as well as healthy controls with similar BMI status. The EVs were further characterized via electron microscopy, western blot and dynamic light scattering. Then, functional analysis was performed on CD8 T cells including apoptosis, cell proliferation, gene expression profiling and cytokine release upon co-incubation with the different EVs. Our results suggest that CRC-derived EVs were able to regulate the CD8 T cells. In some assays, low BMI EVs were functionally different than high BMI EVs. This study highlights the possible difference in the regulatory mechanism of cancer patients-derived EVs, especially on CD8 T cells.

## Introduction

Colorectal cancer (CRC) remains one of the most widely diagnosed cancers worldwide (Bray et al., 2018). Unfortunately, CRC also contributes to a major portion of the number of cancer-associated mortalities (Bray et al., 2018). Several risk factors could lead to the development of CRC such as diet, lifestyle and genetic makeup. One of the other factors influencing the risk of developing CRC is obesity or having a high body mass index (BMI) (Jochem and Leitzmann, 2016; Martinez-Useros and Garcia-Foncillas, 2016). It was shown that individuals with BMI > 25 kg/m^2^ have a higher risk of developing CRC than individuals with a BMI of < 25 kg/m^2^ (Murphy et al., 2000). This association is strong and has been shown in multiple cohort studies (Murphy et al., 2000; Jochem and Leitzmann, 2016). Nevertheless, although higher BMI is associated with higher CRC risk, the survival rate of these patients tends to be better than patients with lower BMI (Aparicio et al., 2018; Shahjehan et al., 2018; Tran et al., 2018). Nevertheless, studies show that BMI did not affect any chemotherapy-based side effects or benefit (Meyerhardt et al., 2003; Sinicrope et al., 2013). In terms of immunotherapy, it has been shown that higher BMI subjects respond better to immune checkpoint inhibitors (Cortellini et al., 2019; Wang et al., 2019). Furthermore, a recent study by Berntsson et al. (2019), suggested that the density of different immune cells within CRC tumors differs based on the BMI status. For instance, it was shown that obesity was associated with a lower percentage of PD-L1 + tumors, but a higher percentage of CD8T cells (Berntsson et al., 2019). Obesity or BMI status likely influences the immune landscape of cancer patients. Nevertheless, our understanding of the link between immunity-obesity-cancer is still preliminary. The effects of treatment in CRC can also be divided based on the metastatic potential of the tumors, this is also in relation to the microsatellite stability of the cancers (Picard et al., 2020). Metastasis is one of the major causes of cancer-related deaths and is found in 20% of CRC cases upon diagnosis (Riihimäki et al., 2016). In terms of immunotherapy, it has been shown that CRC patients with microsatellite instability (MSI) benefit better to this treatment than MSS cancers (Picard et al., 2020).

The efficacy of the immune system is multifaceted, and a lot of factors can influence the immune mechanism. It has been put forward that extracellular vesicles (EVs) can affect the activity of various immune cells such as T cells, natural killer cells and dendritic cells (Whiteside, 2017). EVs are membranous vesicles that are released from cells as circulating entities and can be found in various bodily fluids including serum, urine, and saliva (Whiteside, 2017; Othman et al., 2019). EVs can be divided into several subsets such as microvesicles, exosomes and small EVs (Szatanek et al., 2017). The functional role of EVs is diverse, especially in the tumor setting. For instance, EVs have been shown to have pro- and inflammatory roles when it comes to the tumor microenvironment (Dörsam et al., 2018; Othman et al., 2019). More importantly, tumor-derived EVs have been shown to suppress lymphocyte activity, particularly in CD8 T cells (Muller et al., 2016; Ludwig et al., 2017; Maybruck et al., 2017; Dörsam et al., 2018). For instance, a study by Ludwig et al. (2017) has shown that exosomes derived from head and neck cancers were more immunosuppressive than exosomes from healthy subjects. Concerning BMI or obesity, EVs have been shown to be novel regulators especially when it comes to metabolic-related complications (Kim et al., 2018). Therefore, here we postulate that the presence of cancer as well as the difference in BMI may also impact the function of EVs especially in terms of regulating CD8 T cells.

## Materials and Methods

### Clinical Samples

Serum samples from high BMI-CRC (*n* = 13) and low BMI-CRC (*n* = 15) were obtained from UMBI’s biobank. For the healthy, non-CRC controls, serum samples of high BMI-non-CRC (*n* = 15) and low BMI-non-CRC (*n* = 15) were obtained from The Malaysian Cohort biobank. Samples were obtained from participants with no history of cancer after the second follow-up phase (post 5 years of the initial phase). Serum samples were obtained from healthy non-CRC individuals as well as individuals who were CRC patients with a BMI that falls within the accepted range (high BMI ≥ 25.0 kg/m^2^, low BMI ≥ 18.5– ≤ 25.0 kg/m^2^) since we are using Asian samples (WHO Expert Consultation, 2004; Lim et al., 2017). Blood was also obtained from healthy individuals for lymphocyte isolation. The study was approved by the Research Human Ethics Committee of the Universiti Kebangsaan Malaysia (UKM) (UKM PPI/111/8/JEP-2018-164). All participants gave written informed consent. This study was conducted in concordance with UKM’s standard biosecurity and institutional safety procedures.

### Extracellular Vesicle Isolation

Total EVs were isolated from serum samples using the Total Exosome Isolation (from serum) kit (Invitrogen, United States). Briefly, 50 μl of serum was transferred to a new tube and 0.2 volumes of the Total Exosome Isolation reagent was added. The serum/reagent mixture was mixed well by vortexing and the samples were incubated at 4°C for 45 min. Samples were then centrifuged at 11,000 × *g* for 15 min. The supernatant was then removed and the total EVs were resuspended in 1 × PBS.

### Transmission Electron Microscopy (TEM)

Total EVs were diluted to 1:1000 in PBS. Five microliters of diluted EVs were pipetted onto Formvar-carbon coated EM grids and left aside to allow membranes adsorption for 20 min. The vesicles-coat grids were fixed with 0.6% glutaraldehyde for 4 min and washed twice with distilled water for 1 min each. The grids were stained with 2% uranyl acetate at pH 7 for 5 min. Finally, the grids were viewed using a transmission electron microscope. This method has been previously conducted by our lab (Hon et al., 2019).

### Zeta-Sizer Analysis

Particle size measurement and zeta potential analysis of EVs were performed using the Zetasizer Nano ZS system (Malvern Instruments, Malvern, United Kingdom). EVs were diluted 1: 100 in sterile PBS to a total volume of 1 mL to be loaded into a disposable cuvette for particle size measurement. Data were acquired and analyzed using Zetasizer Software (V7.03) (Malvern Instruments). We have performed this assay according to our previous protocol (Hon et al., 2019).

### Western Blot

Extracellular vesicles lysate was prepared by adding lysis buffer directly to an EV-enriched fraction in PBS and incubated on an orbital shaker at 4°C for 45 min. The mixture was centrifuged, and the supernatant was collected as protein lysate. Protein lysate was quantified using Bradford assay. Fifty μg of proteins were resolved on SDS-polyacrylamide gel electrophoresis (SDS-PAGE) and transferred to membrane. The membrane was blocked with 5% skimmed milk in TBS-T before incubated with primary antibodies at 4°C overnight. Primary antibodies used were mouse monoclonal anti-human TSG101 (Cat# NB200-112 Novus Biologicals, United States. 1:1000 dilution) and anti-human CD9 (Santa Cruz Biotechnology, United States). The membrane was washed with TBS-T before incubated with HRP-conjugated secondary antibodies at room temperature for 1 h. Finally, the blot was washed with TBS-T three times and incubated with Pierce ECL Western Blotting Substrate (Life Technologies, United States) for 10 min. Protein bands were visualized by chemiluminescence using the ChemiDoc MP Imaging System (Bio-Rad, United States).

### Peripheral Blood Mononuclear Cells (PBMCs) Isolation

Fresh whole blood was collected from healthy adult individuals in an anticoagulant-treated EDTA collection tube and PBMCs isolated by Ficoll-Paque (GE Healthcare, United States) gradient centrifugation. Cells were washed in 1 × phosphate-buffered saline (PBS), counted and resuspended in RPMI 1640 medium (Nacalai Tesque, Japan), supplemented with 10% of EV-depleted fetal bovine serum (FBS) and 1% penicillin/streptomycin antibiotics. A portion of cells (50, 000 cells) was stained for analysis by flow cytometry, while the remaining cells underwent positive selection for CD8 T cells.

### CD8 T Cell Isolation and Activation

The highly purified fraction of CD8 T cells were isolated from PBMC cells using the EasySep Human CD8 T Cell Positive Selection Kit II (Stemcell Technologies, Canada), according to the manufacturer’s protocol. A portion of cells was stained for analysis by flow cytometry, while the remaining cells were aliquoted into a T75 flask for T-cell activation. CD8 + T cells were maintained in complete RPMI medium (10% EV-depleted FBS, 1% penicillin/streptomycin), supplemented with 100 IU/ml of interleukin 2 (IL2) (Stemcell Technologies, Canada) and 25 ul of ImmunoCult Human CD3/CD28 T Cell Activator (Stemcell Technologies, Canada) per 1.0 × 10^6^ cells/ml. Cells were incubated at 37°C and 5% CO_2_ for 2–3 days.

### PBMC Pre-sort and Post-sort Staining and Analysis

To view the different populations of cells in the PBMC suspension and the purified CD8 T cells fraction, cells were resuspended in 1 × PBS and transferred to a 5 ml polystyrene round-bottom tube. Cells were stained, on ice, with antibodies to anti-human CD4 (PE), anti-human CD8a (APC) and anti-human CD3 (FITC) (BioLegend, United States) for 30 min in the dark on a rotary shaker. Signals were detected from 1.0 × 105 cell population using the BD FACSVerse flow cytometer (BD Biosciences, United States). The analysis was performed by gating the lymphocytes, followed by the CD3 expression and then the CD4 and CD8 expression. The analysis was performed using FCSExpress (De Novo Software, United States).

### EV Uptake Analysis

To evaluate whether CD8 T cells interacted with EVs, the PKH26 Red Fluorescent Cell Linker Mini Kit (Sigma Aldrich, United States) was utilized to stain the membranes of EVs as per the manufacturer’s protocol. The experiment was performed based on the protocol by Ragni et al. (2017). Briefly, 50 μg of isolated EVs was resuspended in 100 μl Diluent C. Separately, 1.4 μl PKH26 dye was mixed with 300 μl of Diluent C. The two components were combined and gently mixed, and incubated at room temperature for 5 min. The fluorescent labeling reaction was stopped by adding 700 μl of 1% FBS, and the stained EVs were co-cultured with 50,000 CD8 T cells in cell culture medium. Following incubation, cells were harvested and gently centrifuged at 10,000 × *g* for 5 min. The cells were then resuspended in 1 × PBS and analyzed by flow cytometry. EVs without PKH26 dye was incorporated into CD8 T cells as negative controls.

### Establishment of CD8 T Cell Cytokine Profile

Activated CD8 T cells were plated in a 96-well plate (1 × 105 cells/well) in EV-free RPMI. EVs (5 μg/well) isolated from serum high BMI or low BMI CRC or non-CRC patients were added, and co-cultures were incubated for 24 h. Co-cultures containing no EVs (PBS) served as controls. Cell culture supernatant was collected and using the LEGENDplex^TM^ Human CD8/NK Panel (BioLegend, United States), including IL-2, 4, 6, 10, 17A, IFN-γ, TNF-α, soluble Fas, soluble FasL, granzyme A, granzyme B, perforin, and granulysin was quantified by flow cytometry. The analysis was performed via the provided software LEGENDPlex Software v8 (BioLegend, United States).

### Microarray

Activated CD8 T cells were plated and co-cultured with EVs in 96-well plates as previously described. Following 24 h incubation, total RNA was extracted from CD8 T cells using the AllPrep DNA/RNA/miRNA Universal Kit (Qiagen, Germany). RNA was quantified using the NanoDrop 2000C spectrophotometer (Thermo Fisher Scientific, United States) and integrity was analyzed using the Agilent 2100 Bioanalyzer (Agilent Technologies, United States). The total RNA was pooled into groups of three for each of the different groups, except for the LowBMI_CRC and control group, where we were only able to perform analysis on pooled groups of 2. Human gene expression microarray was then performed using the Agilent One-Color SurePrint G3 Human Gene Expression v3 Microarray (Agilent Technologies, United States). The feature extraction, gene expression analysis, and gene ontology analysis were performed using the Altanalyze software (Emig et al., 2010). For pathway enrichment analysis, we used the KEGG database (Kanehisa et al., 2016, 2019) on the Webgestalt platform (Liao et al., 2019).

### Apoptosis V-FITC Apoptosis Assay

Activated CD8 T cells were plated and co-cultured with EVs in 96-well plates as previously described. Following 24 h incubation, apoptosis of CD8 + T cells was measured by flow cytometry using the FITC Annexin V Apoptosis Detection Kit (BD Pharmingen, United States). The experiment was performed with appropriate controls and was gated accordingly. Apoptotic cells were counted based on the early apoptotic population (AnnexinV-FITC + /PI-) and late apoptotic population (AnnexinV-FITC + /PI +).

### CFSE Assay

CD8 T cells were stained with CFSE Dye (Invitrogen, United States) according to the manufacturer’s instructions before co-culturing with the selected EVs. After 3 days of incubation, the cells were subjected to flow cytometry (BD, United States). The analysis of cell proliferation was performed using the ModFit software (Veriti Software House, United States).

### CIBERSORT Analysis

We obtained clinical data and gene expression data of CRC patients from the TCGA database^1^. The samples were divided based on the BMI status, by using the height and weight information obtained from the clinical data. The values from the RNA-Seq data were then entered into CIBERSORT (Newman et al., 2019). The CIBERSORT analysis was performed using the LM22 immune genes as a reference, and other default parameters (Chen et al., 2018). The samples were filtered based on *p* < 0.05 and we performed the Kruskal–Wallis non-parametric test.

### Immune Cell Infiltration Assay

We performed the immune cell infiltration assay using 3D spheroids based on the protocol by Herter et al. (2017) and Courau et al. (2019). Briefly, 3D spheroids of HT-29 cells were generated by seeding 1500 cells/well in a 96 well plate covered with agarose. PBMC from healthy volunteers were obtained and pre-treated with the following group of EVs, Non-CRC-HighBMI (*n* = 9), Non-CRC-LowBMI (*n* = 9), CRC-High-BMI (*n* = 9), and CRC-Low-BMI (*n* = 9) overnight. The PBMC were then transferred to day 3 spheroids and left to incubate for 24 h. Afterward, the immune cells from the supernatant were harvested and pooled into groups of three for each treatment and were considered as the (= OUT) population. The spheroids were washed with ice-cold PBS and trypsinized. The disintegrated spheroids were also pooled into groups of three for each treatment and were considered as the (= IN) population. Both the OUT and IN population were stained with CD3 and CD8 fluorochrome-conjugated antibodies and subjected to flow cytometry.

### Statistical Analysis

All data were presented as mean ± standard deviation (SD). Statistical analysis was performed using GraphPad Prism (version 6) as unpaired *t*-test or one-way ANOVA. Flow analyses were performed with BD FACSuite Software Application (V1.05.3841) (BD Biosciences, United States) and cytokine profile analyses were performed with LEGENDplex Data Analysis Software (BioLegend, United States). A *p*-value of ≤ 0.05 was considered statistically significant.

## Results

### Characterization of EVs

**FIGURE 1:**
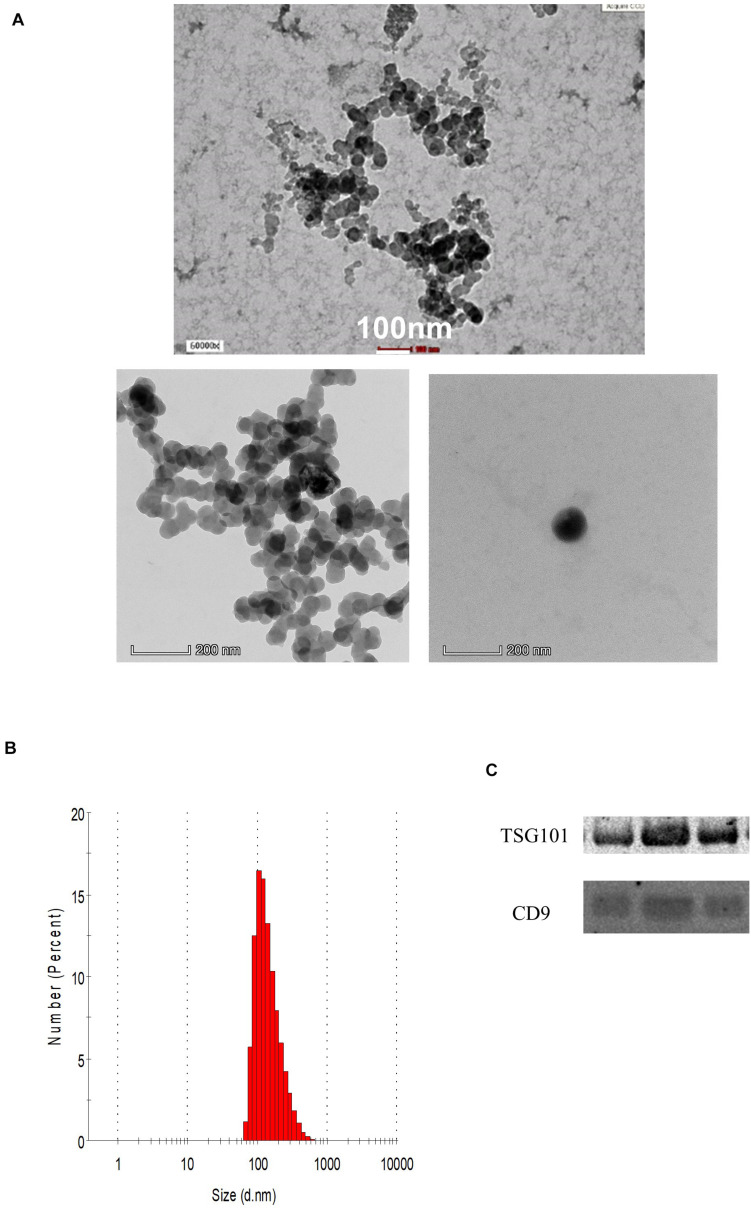
**(A)** Representative transmission electron microscopy images of the enriched population. **(B)** Representative histogram of the dynamic light scattering (DLS) measurement of the EVs. **(C)** Western blot analysis of representative CRC EVs targeting TSG101 and CD9 proteins.

The demographic data of the samples are listed in Table 1. The EVs isolated from the serum of the samples were round and spherical as shown in the transmission electron microscopy images in Figure 1A. Besides electron microscopy, we also performed measurements via dynamic light scattering (DLS) as shown in Figure 1B. Table 2 depicts the mean and mode diameter of representative EVs within each of the groups. According to Table 2, the LowBMI-Non-CRC EVs had the highest mean and mode diameters (340.4 ± 24.52 and 281.86 ± 28.49 nm, respectively), whereas the LowBMI-CRC EVs had the lowest diameters (214.4 ± 12.55 and 98.49 ± 10.19 nm). Interestingly, there were opposite patterns of size between the CRC and the Non-CRC group if depending on the BMI status. Moreover, we also performed immunoblotting on the isolated EVs to detect specific markers, as evidenced in Figure 1C, all tested EVs expressed the EV-based markers TSG101 and CD9.

**TABLE 1:** Summary of the demographic profile of all samples included in the study.

		CRC		Non-CRC	
		HighBMI (*n* = 13)	LowBMI (*n* = 15)	HighBMI (*n* = 15)	LowBMI (*n* = 15)
**Age ± SD (years)**		66 ± 9.4	59.8 ± 8.2	63.2 ± 10.5	62.2 ± 10.5
Gender (%)	Male	10 (76.9)	12 (80.0)	7 (46.7)	7 (46.7)
	Female	3 (23.1)	3 (20.0)	8 (53.3)	8 (53.3)
Race (%)	Malays	9 (69.2)	7 (46.7)	5 (33.3)	5 (33.3)
	Chinese	4 (30.8)	5 (33.3)	5 (33.3)	5 (33.3)
	Indian	–	3 (20.0)	5 (33.3)	5 (33.3)
Tumor location (%)	Colon	2 (15.4)	4 (26.7)		
	Sigmoid colon	5 (38.5)	2 (13.3)		
	Rectum	2 (15.4)	6 (40.0)		
	Rectosigmoid	4 (30.8)	2 (13.3)		
	Anus	–	1 (6.7)		
Tumor stage (%)	I/II	6 (46.2)	7 (46.7)		
	III/IV	5 (38.5)	7 (46.7)		
	Not stated	2 (15.4)	1 (6.7)		
Tumor type (%)	Adenocarcinoma	13 (100)	15 (100)		

*CRC samples were obtained prospectively from the UKM Endoscopy Unit or UKM Medical Molecular Biology’s Biobank. The Non-CRC samples were obtained from The Malaysian Cohort Biobank.*

**TABLE 2:** DLS measurements of the isolated EVs (*n* = 3) in four groups, both mean and mode diameters were analyzed.

	HighBMI-Non-CRC	LowBMI-Non-CRC	HighBMI-CRC	LowBMI-CRC
Mean diameter (d.nm) (z-avg)	245.1 ± 31.22	340.4 ± 24.52	331.4 ± 7.35	214.4 ± 12.55
Mode diameter (d.nm)	124.15 ± 41.08	281.86 ± 28.49	205.15 ± 21.28	98.49 ± 10.19

### EVs Regulated the Protein-Coding and Non-coding RNAs in Activated T Cells

**FIGURE 2:**
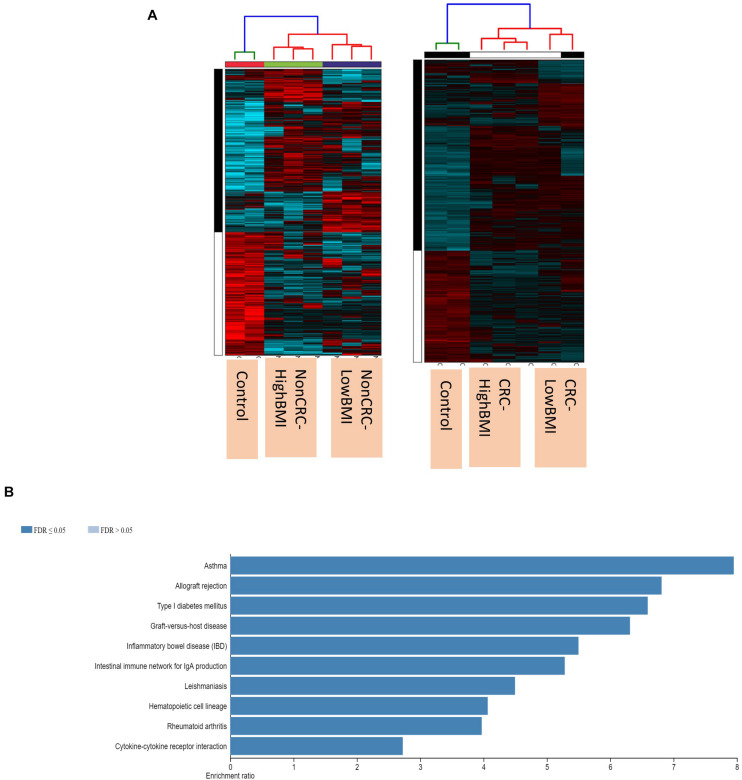
**(A)** Heatmap and clustering of the expression of the Non-CRC and CRC EVs-treated T cells. **(B)** Enriched pathways of the set of genes that were differentially expressed in T cells treated with CRC and Non-CRC EVs.

We performed gene expression analysis in the CD8 + T cells after co-culturing with EVs to observe whether there were any changes at the transcriptomic level (Figure 2). Based on our analysis, there were no significant differentially expressed genes based on the adjusted *p*-value < 0.05. However, there were around 122 dysregulated probes between the HighBMI-non-CRC and LowBMI-non-CRC groups based on the raw *p*-value (<0.05), fold change > 2 and < −2 (Table 3). Nevertheless, there were no significantly enriched pathways detected from this list of genes. Of note, in both comparisons between HighBMI_non-CRC vs. Control, and LowBMI_non-CRC vs. Control the *CCL22* gene was the most upregulated gene. We proceeded with the comparison between HighBMI-CRC and LowBMI-CRC, where no genes were significantly dysregulated based on the adj *p*-value < 0.05. Nevertheless, based on the raw *p*-value (<0.05), the *LPHN2* gene was the most upregulated gene when comparing between HighBMI_CRC vs. LowBMI_CRC as shown in Table 4.

**TABLE 3:** Top 20 up- and down-regulated genes in CD8 T cells treated with EVs from HighBMI_Non-CRC, LowBMI_Non-CRC, and PBS (control).

HighBMI_Non-CRC vs. LowBMI_Non-CRC				HighBMI_Non-CRC vs. Control				LowBMI_Non-CRC vs. Control			
Gene	Fold change	Raw *p*-val	Adj *p*-val	Gene	Fold change	Raw *p*-val	Adj *p*-val	Gene	Fold change	Raw *p*-val	Adj *p*-val
PIGQ	2.652	0.000	0.253	CCL22	5.141	0.002	0.243	CCL22	4.059	0.001	0.312
MBP	2.645	0.001	0.543	RPL17P33	3.467	0.021	0.485	THBD	3.865	0.002	0.359
PLCB1	2.631	0.014	0.949	THBD	3.391	0.019	0.468	NPM2	3.219	0.001	0.312
PLOD2	2.433	0.003	0.851	MON2	3.376	0.000	0.147	CEP44	3.090	0.012	0.584
HMGN2P20	2.424	0.020	0.949	PLEKHA7	3.025	0.008	0.367	CTC-543D15.1	3.028	0.005	0.478
COCH	2.414	0.032	0.949	SOD1P3	2.977	0.012	0.410	CCL2	2.991	0.005	0.476
MAMLD1	2.306	0.002	0.799	ANKRD36C	2.955	0.004	0.301	AP4M1	2.952	0.008	0.526
STRC	2.287	0.002	0.799	AP4M1	2.918	0.001	0.213	IFNLR1	2.949	0.008	0.526
GDI2	2.285	0.003	0.851	SLC16A13	2.888	0.002	0.258	MIAT	2.921	0.005	0.476
SPRR2C	2.269	0.000	0.126	TNNC2	2.868	0.036	0.562	PMEPA1	2.904	0.006	0.492
TRAV12-2	2.267	0.030	0.949	PMEPA1	2.856	0.005	0.322	CC2D1A	2.851	0.036	0.768
ZC3H12D	2.258	0.002	0.793	PRSS36	2.852	0.000	0.147	ORMDL1	2.829	0.029	0.736
TNNC2	2.241	0.042	0.949	CNTRL	2.842	0.000	0.165	PYGO2	2.770	0.002	0.404
LINC00222	2.238	0.009	0.949	TMSB4XP4	2.810	0.007	0.350	FOXH1	2.751	0.000	0.203
DOCK9	2.228	0.009	0.949	AC017116.8	2.763	0.003	0.282	RP5-1063M23.1	2.694	0.018	0.651
PLEKHH3	2.228	0.000	0.253	EP400NL	2.747	0.007	0.350	IL22	2.678	0.000	0.206
C22orf24	2.216	0.007	0.949	FAM210A	2.724	0.004	0.303	SGK494	2.634	0.000	0.238
FAM186B	2.213	0.010	0.949	IVNS1ABP	2.700	0.003	0.269	ANKRD61	2.619	0.023	0.689
MEX3A	2.194	0.000	0.261	PEX2	2.666	0.009	0.372	RP11-72M10.4	2.563	0.009	0.540
LIG4	2.174	0.010	0.949	MKRN3	2.662	0.000	0.168	RP11-689P11.2	2.561	0.013	0.590
CCL2	−3.285	0.000	0.518	CD86	−5.512	0.003	0.275	FCER1G	−5.231	0.040	0.791
ZNF74	−2.407	0.027	0.949	FCER1G	−5.204	0.040	0.583	CD86	−4.218	0.001	0.281
ZBP1	−2.307	0.001	0.568	SULF2	−3.880	0.003	0.287	TTC25	−3.623	0.015	0.616
SOCS2-AS1	−2.292	0.022	0.949	CRTAC1	−3.792	0.001	0.221	SCN4B	−3.206	0.005	0.476
FDXACB1	−2.288	0.003	0.858	HORMAD1	−3.579	0.000	0.148	PTGDS	−2.962	0.013	0.592
PYGL	−2.253	0.000	0.126	RP11-1124B17.1	−3.464	0.018	0.465	GDF15	−2.894	0.003	0.411
DPY19L3	−2.213	0.020	0.949	HBD	−3.121	0.024	0.506	RP11-416N13.1	−2.832	0.009	0.539
LHX9	−2.199	0.012	0.949	PCDH18	−3.092	0.019	0.475	PCDH18	−2.758	0.037	0.773
TSPAN10	−2.170	0.033	0.949	JDP2	−3.068	0.004	0.301	SULF2	−2.666	0.001	0.312
MEST	−2.168	0.005	0.949	MLANA	−3.005	0.000	0.148	RNASE11	−2.611	0.000	0.203
TSHZ2	−2.125	0.027	0.949	CEP170	−2.986	0.005	0.324	CCDC176	−2.588	0.023	0.696
TRAV18	−2.117	0.016	0.949	PAQR3	−2.882	0.001	0.176	RASSF4	−2.579	0.013	0.588
GOLGA6L7P	−2.104	0.018	0.949	ZBP1	−2.857	0.000	0.159	PKIG	−2.527	0.000	0.206
RPL21	−2.099	0.039	0.949	SPRR1B	−2.802	0.010	0.386	PVRL4	−2.511	0.000	0.227
TEX28P2	−2.098	0.000	0.296	CTAGE1	−2.757	0.010	0.394	RAB32	−2.498	0.000	0.206
TSKU	−2.096	0.041	0.949	AC007743.1	−2.753	0.015	0.439	DGKI	−2.493	0.026	0.716
FAM184A	−2.091	0.005	0.949	RP11-134P9.1	−2.722	0.010	0.388	LRRC2	−2.483	0.015	0.620
DNASE1L2	−2.069	0.017	0.949	MAP3K13	−2.676	0.018	0.462	OR4D9	−2.483	0.001	0.276
PLAGL1	−2.069	0.037	0.949	LINC00340	−2.672	0.017	0.457	OR10V1	−2.456	0.007	0.513
FKBPL	−2.067	0.020	0.949	RNASE11	−2.650	0.000	0.139	MAMLD1	−2.436	0.005	0.476

**TABLE 4:** Top 20 up- and down-regulated genes in CD8 T cells treated with EVs from HighBMI_CRC, LowBMI_CRC, and PBS (control).

HighBMI_CRC vs. LowBMI_CRC				LowBMI_CRC vs. Control				HighBMI_CRC vs. Control			
Gene	Fold change	Raw *p*-val	Adj *p*-val	Gene	Fold change	Raw *p*-val	Adj *p*-val	Gene	Fold change	Raw *p*-val	Adj *p*-val
LPHN2	3.028	0.009	0.623	SNRPEP3	8.750	0.001	0.119	WHSC1L1	5.940	0.016	0.564
GTDC2	2.862	0.012	0.698	EEF1A1	7.629	0.000	0.087	CTC-543D15.1	4.869	0.028	0.676
CINP	2.831	0.010	0.647	CTC-543D15.1	7.158	0.001	0.123	TMEM30B	4.265	0.003	0.412
KLHL15	2.777	0.002	0.405	CISD2	5.545	0.010	0.203	C7orf13	4.118	0.007	0.463
CNKSR2	2.752	0.034	0.811	RP11-2C24.5	5.436	0.006	0.192	CD99	4.077	0.000	0.351
KIF3C	2.739	0.021	0.737	FAM105A	4.927	0.000	0.072	ZNF619	3.998	0.000	0.351
PSTK	2.689	0.017	0.718	WHSC1L1	4.696	0.009	0.200	ATHL1	3.982	0.004	0.430
IL17F	2.672	0.000	0.269	RPS15P9	4.444	0.013	0.215	PPIAL4C	3.957	0.001	0.360
EPHX4	2.607	0.001	0.297	ANKRD36C	4.351	0.010	0.201	SNHG14	3.935	0.006	0.462
AC109642.1	2.595	0.004	0.485	RPL7	4.187	0.005	0.187	LINC00574	3.901	0.000	0.351
ZNF29P	2.534	0.002	0.397	TSPYL2	4.053	0.007	0.194	MLTK	3.884	0.033	0.707
MTMR2	2.494	0.040	0.835	TMEM30B	3.937	0.000	0.075	FAM105A	3.872	0.000	0.351
OCLN	2.480	0.000	0.165	ALAS1	3.934	0.000	0.086	FGF9	3.852	0.001	0.351
TRERF1	2.460	0.018	0.718	RTN3	3.858	0.029	0.269	IL1RAP	3.832	0.000	0.351
ZSWIM5	2.448	0.000	0.221	COX20	3.857	0.001	0.121	FAM71E2	3.792	0.000	0.351
KCNQ5	2.431	0.001	0.313	ADAM19	3.824	0.000	0.093	SUFU	3.707	0.000	0.351
CYYR1	2.409	0.025	0.761	DOK7	3.757	0.003	0.181	HBB	3.676	0.024	0.641
PABPC4	2.385	0.030	0.793	FGF9	3.753	0.000	0.102	SUOX	3.671	0.013	0.529
NUTM2B	2.329	0.026	0.769	TXLNG2P	3.741	0.002	0.167	CRYZ	3.633	0.002	0.374
ZHX3	2.311	0.041	0.837	FAM210A	3.691	0.001	0.132	TNFRSF11A	3.603	0.001	0.351
SLC25A25	−3.552	0.002	0.403	PTPRK	−4.464	0.018	0.231	IGLL5	−5.589	0.043	0.766
ALDH1A1	−2.833	0.000	0.136	CALML6	−4.351	0.004	0.184	KIF3C	−4.167	0.003	0.407
FAM178B	−2.666	0.001	0.309	AC011286.1	−4.187	0.000	0.091	CALML6	−4.141	0.017	0.573
HOXA7	−2.627	0.001	0.340	OR6C74	−3.802	0.003	0.181	GS1-122H1.2	−3.750	0.007	0.462
AKAP9	−2.564	0.005	0.507	TMEM52	−3.722	0.006	0.191	PTPRK	−3.687	0.043	0.767
SPANXB2	−2.560	0.002	0.404	SLC25A25	−3.710	0.002	0.169	ZXDA	−3.640	0.004	0.417
TINAG	−2.557	0.005	0.507	CDH9	−3.645	0.000	0.051	GPR75	−3.606	0.006	0.461
INTS2	−2.543	0.024	0.757	GS1-122H1.2	−3.555	0.010	0.203	IFNE	−3.510	0.015	0.552
RP11-545A16.1	−2.516	0.000	0.136	RAD9B	−3.494	0.000	0.079	NUTMF	−3.470	0.001	0.360
EEF1A1P12	−2.489	0.019	0.724	SPP1	−3.466	0.000	0.088	VIT	−3.460	0.006	0.462
RP11-183M13.1	−2.454	0.000	0.187	GPR75	−3.423	0.002	0.165	CYP2B6	−3.408	0.012	0.515
SERHL2	−2.431	0.036	0.824	FAM183A	−3.385	0.001	0.134	CHRNA7	−3.362	0.036	0.723
OR4F21	−2.428	0.000	0.207	IRGC	−3.247	0.006	0.192	IL17F	−3.292	0.002	0.374
OR10V1	−2.401	0.000	0.165	SFRP2	−3.234	0.001	0.155	OR6C74	−3.229	0.026	0.657
OR1J4	−2.388	0.026	0.769	HIF3A	−3.200	0.000	0.052	CDH9	−3.186	0.001	0.361
AF131215.4	−2.378	0.001	0.297	MCF2L	−3.137	0.021	0.243	PSTK	−3.163	0.017	0.576
MBP	−2.365	0.001	0.310	ETV1	−3.117	0.008	0.197	PPP1R12B	−3.109	0.006	0.459
CTD-2526L21.2	−2.340	0.001	0.305	BICC1	−2.986	0.009	0.200	BICC1	−3.100	0.035	0.721
RP11-308D16.4	−2.335	0.000	0.245	OR5B2	−2.959	0.001	0.149	ETV1	−3.092	0.022	0.632
IGHV3-30	−2.332	0.000	0.150	RSPH1	−2.948	0.000	0.051	C7orf72	−3.080	0.006	0.462

**FIGURE 3:**
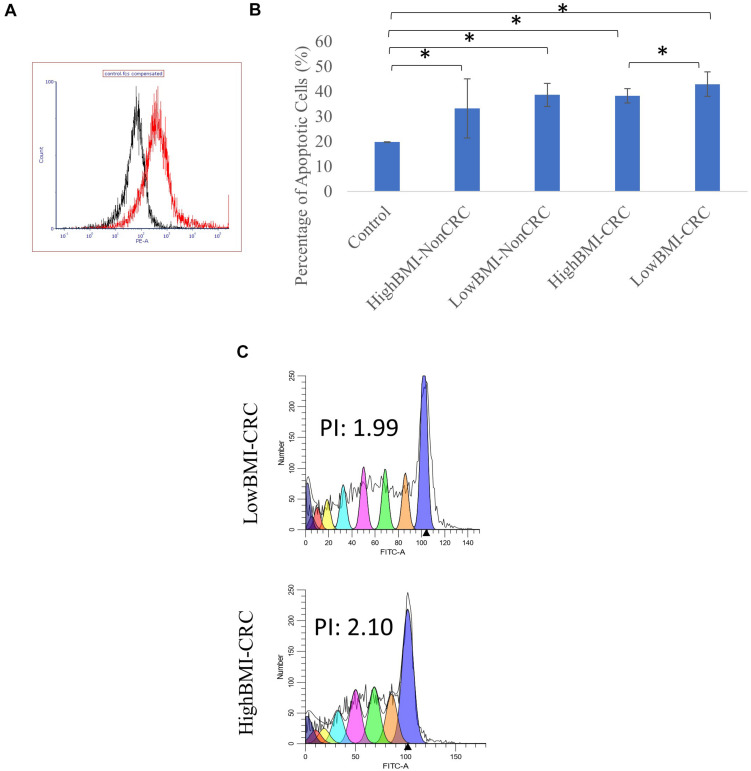
**(A)** Uptake of PKH26 dye by the EVs as evaluated by flow cytometry analysis. **(B)** Normalized fold change of the apoptotic rate in the CD8T cells upon co-incubation with different groups of EVs; HighBMI-Non-CRC, LowBMI-non-CRC, HighBMI-CRC, and LowBMI-non-CRC. **(C)** Representative CFSE analysis in the CD8T cells co-cultured with CRC-EVs as evaluated by flow cytometry, HighBMI-CRC (*n* = 4), LowBMI-CRC (*n* = 4). PI, proliferation index. * indicates statistical significance at *p* < 0.05.

Interestingly, when comparing between CRC and Non-CRC, there were over 2000 probes that were significantly regulated (adj *p*-value < 0.05) (Table 5). Some of the most enriched pathways (FDR < 0.05) that were identified from this set of genes include cytokine-cytokine receptor interaction, inflammatory bowel disease and intestinal immune network for IgA production (Figure 3B). Besides regulating the protein-coding RNA, the EVs were also able to alter the expression of non-coding RNAs (ncRNAs) in the T cells as well. For instance, LINC00222 was upregulated in HighBMI_Non-CRC as compared to LowBMI_Non-CRC (raw *p*-value < 0.05, fold change > 2). Interestingly, some of the identified lncRNAs were upregulated in the CRC-treated T cells as compared to the non-CRC groups such as *MIAT, MEG3*, and *LINC00426.* Whereas certain lncRNAs were downregulated, such as *PVT1* and *LINC00520.* Nevertheless, careful interpretation of this data is needed as the T cells used were different between the CRC and non-CRC EVs and this could contribute to the high differences.

**TABLE 5:** Top 20 up- and down-regulated genes in CD8 T cells treated with EVs from Non-CRC, CRC (regardless of BMI), and PBS (control).

CRC vs. Non-CRC				CRC vs. Control				Non-CRC vs. Control			
Gene	Fold change	Raw *p*-val	Adj *p*-val	Gene	Fold change	Raw *p*-val	Adj *p*-val	Gene	Fold change	Raw *p*-val	Adj *p*-val
ZNF683	20.593	0.000	0.000	CTC-543D15.1	5.073	0.000	0.212	IL2	6.365	0.043	0.347
STMND1	17.068	0.000	0.000	EEF1A1	3.377	0.029	0.434	B3GNT5	5.626	0.049	0.359
KRT1	12.728	0.000	0.000	LINC00426	3.339	0.034	0.447	CTTN	5.131	0.046	0.353
CMKLR1	12.596	0.000	0.000	CORO1B	3.105	0.005	0.389	PDGFA	4.806	0.050	0.360
FCRL6	10.258	0.000	0.000	PTPN3	3.033	0.002	0.334	CD200	4.687	0.016	0.285
FCRL6	9.911	0.000	0.000	Sep-09	3.001	0.011	0.394	NR4A2	4.391	0.015	0.284
RAB37	9.204	0.000	0.000	SNHG14	2.930	0.001	0.286	CCL22	4.143	0.000	0.027
NMUR1	8.288	0.000	0.000	TMSB15A	2.913	0.000	0.233	CISH	4.128	0.012	0.271
PTPRN2	7.916	0.000	0.000	KRTAP19-8	2.911	0.001	0.234	CISD2	3.915	0.006	0.240
PTGDS	7.567	0.000	0.000	ITGB2	2.899	0.001	0.258	SNRPEP3	3.805	0.020	0.300
CD244	7.308	0.000	0.000	GNB5	2.897	0.026	0.429	SLC16A1	3.794	0.007	0.248
SPON2	7.219	0.000	0.000	FAM105A	2.836	0.006	0.390	ANKRD36C	3.655	0.001	0.167
LINC00426	7.169	0.000	0.000	ATHL1	2.820	0.001	0.298	GOLIM4	3.654	0.021	0.303
FGR	7.072	0.000	0.000	RP11-539L10.2	2.794	0.032	0.444	C17orf58	3.606	0.033	0.329
RAB37	6.929	0.000	0.000	AC010984.3	2.767	0.004	0.372	FAM210A	3.592	0.000	0.138
RASGRP2	6.833	0.000	0.000	PTPRN2	2.759	0.026	0.428	MB	3.570	0.024	0.309
RP11-539L10.2	6.807	0.000	0.000	RLN1	2.742	0.001	0.234	CTC-543D15.1	3.564	0.000	0.120
KLF2	6.575	0.000	0.000	CCR2	2.728	0.029	0.434	IRF8	3.547	0.049	0.359
DAPK2	6.284	0.000	0.000	OTOP1	2.716	0.004	0.374	XIRP1	3.536	0.000	0.130
FRY	6.055	0.000	0.000	NRADDP	2.711	0.000	0.079	CCL17	3.503	0.044	0.350
IL3	−45.855	0.000	0.000	IL3	−7.944	0.047	0.469	CMKLR1	−5.066	0.016	0.285
IL2	−38.913	0.000	0.000	INSM1	−7.444	0.026	0.429	PTGDS	−4.817	0.001	0.154
INSM1	−27.885	0.000	0.000	CCL17	−5.726	0.019	0.407	FCRL6	−4.799	0.002	0.203
CTTN	−25.622	0.000	0.000	IL31	−5.682	0.019	0.407	ZNF683	−4.730	0.026	0.315
B3GNT5	−22.520	0.000	0.000	IFIT1	−5.162	0.015	0.397	FCRL6	−4.089	0.015	0.282
CCL17	−20.063	0.000	0.000	DOK5	−4.719	0.011	0.394	AC007743.1	−4.018	0.006	0.241
IL31	−16.082	0.000	0.000	IL17F	−4.321	0.015	0.398	CD244	−3.796	0.005	0.235
SCHIP1	−14.379	0.000	0.000	SLIT3	−4.044	0.009	0.393	AC011286.1	−3.523	0.014	0.280
IL24	−13.591	0.000	0.000	GDF15	−3.801	0.010	0.393	FGR	−3.432	0.016	0.285
CCL18	−13.460	0.000	0.000	TMEM54	−3.769	0.012	0.394	NMUR1	−3.365	0.026	0.316
CISH	−12.943	0.000	0.000	IL9	−3.632	0.047	0.469	RAB37	−3.199	0.029	0.323
IL9	−12.635	0.000	0.000	RGS16	−3.569	0.032	0.443	RPS3	−3.167	0.018	0.292
CD200	−11.433	0.000	0.000	PHEX	−3.554	0.029	0.434	GLTSCR1	−3.163	0.015	0.285
DOK5	−11.105	0.000	0.000	PPP1R14C	−3.550	0.000	0.142	CD86	−3.117	0.001	0.177
IFIT1	−10.806	0.000	0.000	LHX1	−3.549	0.001	0.241	KLF2	−3.088	0.022	0.306
PHLDA2	−10.486	0.000	0.000	CALD1	−3.524	0.043	0.459	NAALADL2	−3.070	0.004	0.225
ACSM2B	−9.248	0.000	0.000	SFRP2	−3.497	0.000	0.074	SLC5A2	−3.031	0.009	0.259
CD200	−8.936	0.000	0.000	KIF1A	−3.413	0.027	0.430	SPON2	−3.029	0.013	0.276
RGS16	−8.655	0.000	0.000	SLC6A9	−3.411	0.002	0.339	PACSIN1	−3.005	0.040	0.343
DIRAS3	−8.584	0.000	0.000	PTPRK	−3.370	0.003	0.342	DAPK2	−3.005	0.010	0.263

### CRC-LowBMI EVs Stimulate an Increase in CD8 T Cell Apoptosis and Regulated the Cytokine Profile

Prior to determining the biological function of EVs in CD8 T cells, the uptake of EVs by CD8 T cells was first assessed. Flow cytometric analysis of the PKH26 dye uptake demonstrated that the EVs interacted with the T cells (Figure 3A). A study by Muller et al. (2017), demonstrated that Treg cells did not internalize tumor-derived exosomes, but rather interacted via cell surface signaling. It was shown that through this interaction, exosomes were able to induce apoptosis in T cells (Muller et al., 2016, 2017). Therefore, we proceeded with the apoptosis analysis. Using the Annexin V-FITC apoptotic assay, cell death in CD8 T cells was evaluated following co-culture with EVs as depicted in Figure 3B. It was determined that CD8 T cells co-cultured with LowBMI-CRC EVs had an increased cell death (42.89%) as compared to the other groups. There was a significant difference (*p* < 0.05) in cell death between the LowBMI-CRC and HighBMI-CRC EVs. However, no significant changes were observed in CD8 T cells co-cultured with non-CRC EVs. Additionally, there was also no significant difference in cell death when comparing between CRC and non-CRC EVs. Nevertheless, regardless of the disease or BMI status, the EVs managed to induce cell death as compared to the untreated CD8 T cells. Since there were differences in terms of apoptosis in the T cells treated with CRC EVs, we wanted to see whether the same effect could be seen in the cell proliferation CFSE assay. As shown in Figure 3C, the LowBMI-CRC EVs had a lower proliferation index than the HighBMI-CRC EVs, though no significance was observed.

**FIGURE 4:**
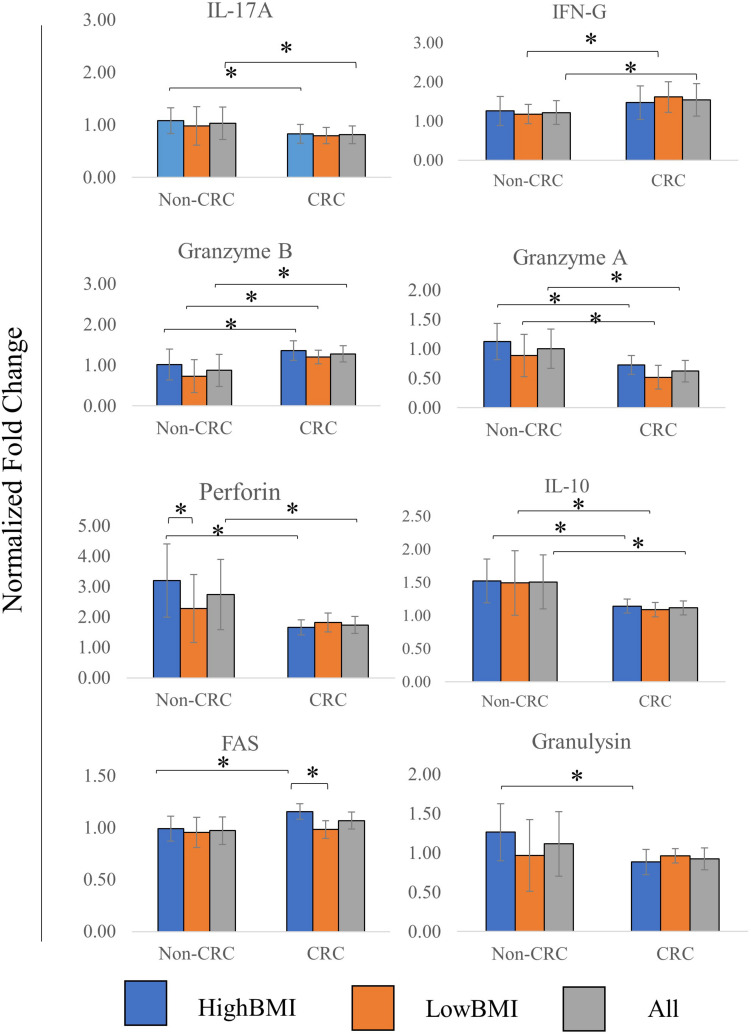
Normalized fold change ± SD of the secreted cytokines analyzed using multiparametric flow cytometry for each group. Experimental values were normalized against cytokines released from untreated T cells. The “All” group is the combination of CRC and Non-CRC regardless of BMI status (*n* = 8, for each group). ANOVA analysis was performed for each of the cytokines and statistical significance was set at *p* < 0.05. * indicates statistical significance at *p* < 0.05.

The level of several related cytokines in CD8 T cells co-cultured with EVs from high BMI or low BMI CRC and non-CRC patients were quantified (Figure 4). For IL-17A, elevated expression of this cytokine was observed in the T cells incubated with Non-CRC EVs, significance (*p* < 0.05) was observed between the HighBMI-non-CRC and HighBMI-CRC (*p* = 0.023), as well as for the overall Non-CRC against CRC (*p* = 0.007). Similarly, this observation was also seen in the release of perforin, granzyme A, IL-10 and granulysin. For perforin, there was a significant difference between the HighBMI-non-CRC and HighBMI-CRC (*p* = 0.0019). Interestingly for perforin, the level of release was higher in the HighBMI-Non-CRC than the LowBMI-Non-CRC group (*p* = 0.001). For granulysin, only the highBMI group was different between the Non-CRC and CRC groups (*p* = 0.039). Moreover, for IL-10 and granzyme A, the difference of expression was observed between the HighBMI and LowBMI in CRC and Non-CRC EVs respectively (granzyme A: *p* = 0.005 and *p* = 0.03, IL-10: *p* = 0.01 and *p* = 0.03 respectively) As for IFN-G, T cells incubated with CRC-EVs had a higher release of this cytokine as compared to the non-CRC-EVs (*p* = 0.01). The same pattern of expression was also found in the release of granzyme B (*p* = 0.001). Subsequently, for the release of Fas, the highBMI-CRC had a higher release than the lowBMI-CRC group (*p* = 0.001).

It is worth noting that in our transcriptomic analysis, the gene expression levels of several cytokines were also significantly regulated (adj *p* < 0.05 and fold change > 2). Although there seems to be no difference between the different BMI status, we observed an interesting pattern when comparing CRC and non-CRC EVs. For instance, the mRNA expression of both IL-2 and IL-3 were significantly downregulated in the T cells treated with CRC EVs as compared to the non-CRC EVs (Table 5). Other cytokines such as IL-9 and IL-31 were also downregulated in T cells treated with CRC EVs.

### T Cell Infiltration of CD8 T Cells Was Regulated Upon Treatment With EVs

**FIGURE 5:**
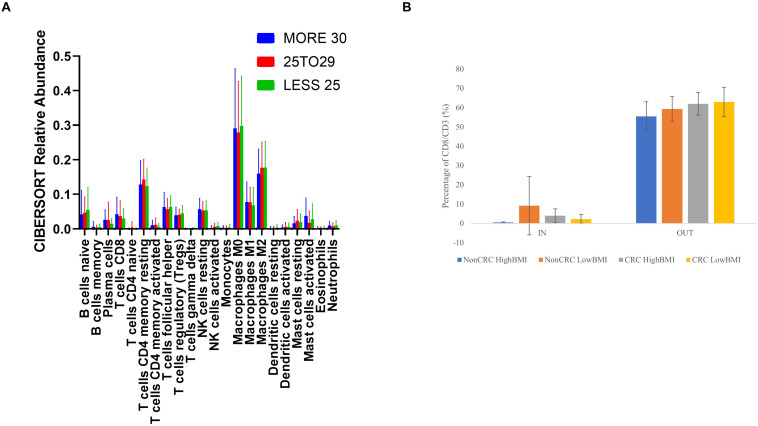
**(A)** Mean ± SD fractions of tumor-infiltrating immune cells based on different BMI status from the TCGA cohort using CIBERSORT analysis HighBMI: > 30 kg/m^2^, Normal BMI: 29.9–25 kg/m^2^, LowBMI:25–18.5 kg/m^2^. **(B)** Percentage of T cells in the IN and OUT population of the assay.

We performed an analysis of the composition of immune cells within the RNA-Seq data using the TCGA cohort. Upon segregation of the samples based on the BMI status, we entered the gene expression data into CIBERSORT. Based on the analysis, there were no significant differences in terms of the composition of immune cells between three sets of BMI (>30, 29.99–25, and 25–18.5 kg/m^2^). Nevertheless, although no significant difference was observed, we did see an elevated level of abundance in the CD8 T cell population (1.44 fold change against the 25–28.5 kg/m^2^ group), and macrophage M1 population (1.13 fold change against the 25–18.5 kg/m^2^ group) in the > 30 kg/m^2^ group (Figure 5A). Concomitantly, there was a reduction in the activated natural killer cells populations (0.25 fold change against the 25–18.5 kg/m^2^ group) in the highest BMI group. To further analyze whether the EVs isolated from the samples were able to affect the ability of T cells infiltration, we performed the 3D spheroid infiltration After 24 h of treatment with the EVs (Figure 5B), the percentage of T cells infiltrating the HT-29 spheroids was measured. As shown in Figure 5B, CD8 T cells treated with LowBMI-Non-CRC EVs had the highest percentage of infiltration as compared to the other EVs, although no statistical significance was observed. Additionally, there were also no significant differences between the HighBMI-CRC and LowBMI-CRC group.

## Discussion

The “obesity paradox” is an intriguing concept in linking adiposity and cancer prognosis (Park et al., 2018; Ujvari et al., 2019). Interestingly, there seems to be a link between obesity and the tumor microenvironment, particularly the immune parameters and mechanisms, especially in CD8 T cells (Turbitt et al., 2020). The strong association between BMI and immune response has been well-established, particularly in immunotherapy-based treatments (Ilavská et al., 2012; Canter et al., 2018). The release of soluble factors such as cytokines or hormones has been known to be regulated by the presence of obesity/adiposity or high BMI (Schmidt et al., 2015; Del Cornò et al., 2016). Nevertheless, with regard to EVs, no studies have been conducted to delineate the effects of BMI, much less CRC-related BMI toward the tumor microenvironment. Indeed, our study has shown that there may be subtle differing effects of the BMI and the presence of cancer on the functional roles of EV, especially in mediating response to CD8 T cells.

Extracellular vesicles are defined as membrane encapsulated vesicles that are released from the cells and into the bodily fluids. In the EV subset, exosomes are known to be sized between 30 and 150 nm, whereas microvesicles are sized between 100 and 1000 nm (Doyle and Wang, 2019). Our isolated EVs are sized on average roughly between 201.86 and 364.94 nm as measured using DLS method. Other methods of measurement, for instance using nanoparticle tracking analysis, should also be conducted to further confirm the size of the isolated EVs, however this instrument is not available to us at the time of the study (Doyle and Wang, 2019). Interestingly, in the isolated EVs within the non-CRC group, the lowBMI EVs had a larger size, this was opposite than what was found in the CRC group where the highBMI EVs were larger. In a study by Enjeti et al. (2017), it was shown that the BMI status and lipid profile may affect the released microvesicles in terms of marker and functional effects.

The functional roles of EVs in the tumor microenvironment have been previously established in several cancers, such as melanoma (Sharma et al., 2020), head and neck cancer (Ludwig et al., 2017; Maybruck et al., 2017), pancreatic cancer (Fan et al., 2018), and breast cancer (Wen et al., 2016). In relation to CD8 T cells, several recent studies have shown that EVs from cancer patients as well as from cancer cell lines were able to suppress CD8 T cells by inducing apoptosis and reducing cell proliferation (Muller et al., 2016; Ludwig et al., 2017; Sharma et al., 2020). For instance, a study by Maybruck et al. (2017) showed that tumor-derived exosomes suppressed CD8 T cells. Our results indicate that low BMI-CRC EVs induced more apoptosis in CD8 T cells than the high BMI-CRC EVs, however, no significance was observed in the non-CRC groups. We wanted to see whether this same effect could also be seen in terms of proliferation, so we performed the CFSE based assay. As expected, the number of proliferating cells in T cells co-incubated with low BMI-CRC EVs was also reduced although no significance was observed. Studies have shown that EVs carrying Fas/FasL were able to mediate apoptosis in immune cells (Abusamra et al., 2005). In this study, there was a difference in the apoptotic rate of the CD8 T cells, but we have not confirmed the mechanism of apoptosis. Apart from that, the detection of soluble Fas (sFas) was indeed higher in highBMI-CRC group. It was previously shown that the release of sFas could inhibit fas-mediated apoptosis (Cheng et al., 1994; Cascino et al., 1995; Volpe et al., 2016). Therefore, we postulate that the EVs could stimulate the release of higher sFas in highBMI-CRC group, and subsequently lead to a lower percentage of cell death than lowBMI-CRC group. Nevertheless, further confirmation of this proposed mechanism is needed.

Multiple studies have shown that obesity or patients with high adiposity often have T cell dysfunction either in terms of T cell metabolism, T cell exhaustion, or overall T cell reactivity (Aguilar and Murphy, 2018; Wang et al., 2019). The presence of chronic inflammation as a result of obesity have exacerbated the production of inflammation-related cytokines (Schmidt et al., 2015). As evidenced by our results, the release of several cytokines differed between the different groups. IL-17A, IL-10, Granzyme A, Perforin and Granulysin, for instance, were generally higher in the non-CRC EVs-treated CD8T cells. However, the levels of other effectors such as IFN-G and Granzyme B were regulated differently. Interestingly, it has been shown that the release of IFN-G from CD8 T cells increases its cytotoxic activity and thus reducing tumorigenesis (Bhat et al., 2017; Ni and Lu, 2018). Nevertheless, recent studies have shown that IFN-G possessed dual roles in regulating cancer. Apart from being involved in immunosurveillance, IFN-G was shown to promote tumorigenesis via immunoevasion (Mojic et al., 2017; Ni and Lu, 2018). Although various factors could influence the release of IFN-G, it is interesting to note that the EVs from CRC patients were able to elicit higher amounts of IFN-G than the non-CRC EVs. Interestingly, when comparing CRC and Non-CRC-treated T cells, the RNA level of IL-2 and IL-3 were significantly regulated. IL-2 has been shown to be able to regulate the activity of CD8 T cells in terms of memory and activation (Mallard et al., 2004; Kalia and Sarkar, 2018; Maimela et al., 2019).

Besides the apoptotic effects, the tumor-infiltrating abilities of the T cells were also investigated using a modified 3D spheroid model (Courau et al., 2019). Prior to that, we wanted to get an overview of the immune cell composition with regards to the BMI status using bulk RNA-Seq data. Based on our CIBERSORT analysis using TCGA data, although no significance was observed, there was an elevated level of CD8 T cells in the higher BMI group. In a study by Hanyuda et al. (2016), they found that there were no significant association between CRC risk and BMI based on the density of T cells, including CD8 T cells. Therefore, we wanted to see whether the EVs isolated from these different groups could affect the ability of lymphocytes in infiltrating tumor spheroids. Interestingly, lymphocytes treated with CRC-HighBMI EVs were able to penetrate the tumor spheroids at higher concentrations than the CRC-LowBMI EVs, albeit no significance was observed. Interestingly, in the non-CRC groups, the low BMI group had higher infiltration than the high BMI EVs. This also shows that non-CRC EVs could be more effective at inducing lymphocyte infiltration than CRC EVs, and interestingly, the effects of BMI were opposite in the two groups. This indicates that the “BMI effect” may also be dependent on the health status of the subjects. However, this preliminary results still need further confirmation in a more translational model.

Based on our transcriptomic analysis, there were differences in the regulation of lncRNAs between CRC and non-CRC EV-treated T cells. Although the information on the regulation of lncRNA within the CD8 T cells transcriptomic profile is still limited, it has been shown that lncRNAs are able to function as immunomodulators (Zhou et al., 2019). For instance, in a recent study, it was discovered that the lncRNA *Morrbid* was able to regulate CD8 T cell survival upon viral infection (Kotzin et al., 2019). In a different study, Ji et al. (2018), showed that lnc-tim3 was able to regulate CD8 T cell exhaustion in hepatocellular carcinoma. In this study, we discovered that upon co-incubation with different EVs, the regulation of lncRNA within the T cells was also affected but no further in-depth analysis was conducted. Collectively, there is still a lot of uncharted territories when it comes to lncRNA regulation in CD8T cells and more future studies are needed to fully elucidate its role especially in tumor-related immunity.

## Conclusion

For this study, we used BMI as a measurement to differentiate overweight and lean patients. Nevertheless, it has been shown that the percentage of adipose tissue is a more accurate determinant in categorizing obesity (Martinez-Useros and Garcia-Foncillas, 2016; Caan et al., 2018). This is the limitation of our study, the only information available to us at the time of collection for the CRC samples is the height and weight of the patients before obtaining the samples. Further correlation between adiposity and tumor immunity could strengthen the outcome of this study. Apart from the BMI factor, the status of microsatellite stability also plays an important role in terms of treatment selection and overall prognosis (Boland and Goel, 2010). Moreover, it has been shown that patients with microsatellite stable (MSS) cancers do not benefit from immunotherapy as microsatellite instable (MSI) cancers (Emambux et al., 2018). In fact, in a recent study, it was shown that the dynamics of the T-cells phenotype differ between MSS and MSI cancers (Di et al., 2020). Future studies should be conducted to correlate the status of microsatellite stability with the functional effects of EVs.

On top of that, based on the distribution of our samples, most of the CRC samples were derived from male patients, thus creating a gender imbalance in the overall population. A study by Enjeti et al. (2017), has shown that there were no significant differences in the level of microvesicles between males and females, however, there were functional differences in terms of procoagulant activity. We are aware of this limitation, and this should be further considered for future downstream analysis and careful interpretation of this data. Apart from that, the CRC samples were obtained from patients with different stages of CRC. This could also affect the interpretation of this data. Furthermore, we isolated the total pre-enriched fraction of EVs from the serum instead of focusing on either small EVs or marker-specific EVs such as CD63 + or CD81 + population. We wanted to see whether as a whole population of EVs could affect the activity of the lymphocytes, although there could be a specific population within the EVs that contribute to the immune function. Further purification or isolation of specific sub-group of EVs such as small EVs could also enhance our understanding of the mechanism of EVs-mediated immunity. On top of that, we isolated the EVs from the systemic circulation using blood derived from the patients. We are unable to determine which cells do the EVs originated from. This should be further elucidated in future experiments to determine precisely which cells contribute toward the immunomodulating EVs. Overall, the results we presented here are a “proof of concept” that BMI could be a factor in influencing the roles of EVs and this may eventually lead to the suppression of tumor immunity. Nevertheless, further in-depth analysis is needed to concretely establish the relationship between BMI, cancer and immune suppression. On top of that, further studies on the role of EVs toward other immune cells such as CD4 and natural killer cells, and how they regulate the effector roles of these cells should also be investigated.

## Data Availability Statement

The datasets presented in this study can be found in online repositories. The names of the repository/repositories and accession number(s) can be found below: “https://www.ncbi.nlm.nih.gov/, GSE152508 and https://www.ncbi.nlm.nih.gov/geo/query/acc.cgi?acc=GSE152508.”

## Ethics Statement

The studies involving human participants were reviewed and approved by Universiti Kebangsaan Malaysia’s Ethical Committee on Human Research. The patients/participants provided their written informed consent to participate in this study.

## Author Contributions

NA, NO, NR, and NB: formal analysis. NA: funding acquisition. NO, NR, NB, SN, and JC: investigation. NA, NO, and SN: methodology. LM, ZA, and RJ: resources. NA and RJ: supervision. NA and NO: writing – original draft. RJ: writing – review and editing. All authors contributed to the article and approved the submitted version.

## Conflict of Interest

The authors declare that the research was conducted in the absence of any commercial or financial relationships that could be construed as a potential conflict of interest.

## Footnotes

1 https://www.cancer.gov/tcga

## References

[B1] Abusamra A. J. Zhong Z. Zheng X. Li M. Ichim T. E. Chin J. L. (2005). Tumor exosomes expressing Fas ligand mediate CD8+ T-cell apoptosis. *Blood Cells Mol. Dis.* 35 169–173. 10.1016/j.bcmd.2005.07.001 16081306

[B2] Aguilar E. G. Murphy W. J. (2018). Obesity induced T cell dysfunction and implications for cancer immunotherapy. *Curr. Opin. Immunol.* 51 181–186. 10.1016/j.coi.2018.03.012 29655021 PMC6338436

[B3] Aparicio T. Ducreux M. Faroux R. Barbier E. Manfredi S. Lecomte T. (2018). Overweight is associated to a better prognosis in metastatic colorectal cancer: a pooled analysis of FFCD trials. *Eur. J. Cancer* 98 1–9. 10.1016/j.ejca.2018.03.031 29807237

[B4] Berntsson J. Eberhard J. Nodin B. Leandersson K. Larsson A. H. Jirström K. (2019). Pre-diagnostic anthropometry, sex, and risk of colorectal cancer according to tumor immune cell composition. *Oncoimmunology* 8:e1664275. 10.1080/2162402X.2019.1664275 31741761 PMC6844316

[B5] Bhat P. Leggatt G. Waterhouse N. Frazer I. H. (2017). Interferon-γ derived from cytotoxic lymphocytes directly enhances their motility and cytotoxicity. *Cell Death Dis.* 8:e2836. 10.1038/cddis.2017.67 28569770 PMC5520949

[B6] Boland C. R. Goel A. (2010). Microsatellite instability in colorectal cancer. *Gastroenterology* 138 2073–2087.e3. 10.1053/j.gastro.2009.12.064 20420947 PMC3037515

[B7] Bray F. Ferlay J. Soerjomataram I. Siegel R. L. Torre L. A. Jemal A. (2018). Global cancer statistics 2018: GLOBOCAN estimates of incidence and mortality worldwide for 36 cancers in 185 countries. *CA Cancer J. Clin.* 68 394–424. 10.3322/caac.21492 30207593

[B8] Caan B. J. Cespedes Feliciano E. M. Kroenke C. H. (2018). The importance of body composition in explaining the overweight paradox in cancer-counterpoint. *Cancer Res.* 78 1906–1912. 10.1158/0008-5472.CAN-17-3287 29654153 PMC5901895

[B9] Canter R. J. Aguilar E. Wang Z. Le C. Khuat L. Dunai C. (2018). Obesity results in higher PD-1-mediated T-cell suppression but greater T-cell effector functions following blockade. *J. Clin. Oncol.* 36 65–65. 10.1200/JCO.2018.36.5_suppl.65

[B10] Cascino I. Fiucci G. Papoff G. Ruberti G. (1995). Three functional soluble forms of the human apoptosis-inducing Fas molecule are produced by alternative splicing. *J. Immunol.* 154 2706–2713. 7533181

[B11] Chen B. Khodadoust M. S. Liu C. L. Newman A. M. Alizadeh A. A. (2018). Profiling tumor infiltrating immune cells with CIBERSORT. *Methods Mol. Biol.* 1711 243–259. 10.1007/978-1-4939-7493-1_12 29344893 PMC5895181

[B12] Cheng J. Zhou T. Liu C. Shapiro J. Brauer M. Kiefer M. (1994). Protection from Fas-mediated apoptosis by a soluble form of the Fas molecule. *Science* 263 1759–1762. 10.1126/science.7510905 7510905

[B13] Cortellini M. Bersanelli S. Buti Cannita K. Santini D. Perrone F. Giusti R. (2019). A multicenter study of body mass index in cancer patients treated with anti-PD-1/PD-L1 immune checkpoint inhibitors: when overweight becomes favorable. *J. Immunother. Cancer* 7:57. 10.1186/s40425-019-0527-y 30813970 PMC6391761

[B14] Courau T. Bonnereau J. Chicoteau J. Bottois H. Remark R. Assante Miranda L. (2019). Cocultures of human colorectal tumor spheroids with immune cells reveal the therapeutic potential of MICA/B and NKG2A targeting for cancer treatment. *J. Immunother. Cancer* 7:74. 10.1186/s40425-019-0553-9 30871626 PMC6417026

[B15] Del Cornò M. D’Archivio M. Conti L. Scazzocchio B. Varì R. Donninelli G. (2016). Visceral fat adipocytes from obese and colorectal cancer subjects exhibit distinct secretory and ω6 polyunsaturated fatty acid profiles and deliver immunosuppressive signals to innate immunity cells. *Oncotarget* 7 63093–63105. 10.18632/oncotarget.10998 27494857 PMC5325349

[B16] Di J. Liu M. Fan Y. Gao P. Wang Z. Jiang B. (2020). Phenotype molding of T cells in colorectal cancer by single-cell analysis. *Intern. J. Cancer* 146 2281–2295. 10.1002/ijc.32856 31901134

[B17] Dörsam B. Reiners K. S. von Strandmann E. P. (2018). Cancer-derived extracellular vesicles: friend and foe of tumour immunosurveillance. *Philos. Trans. R. Soc. Lond. B Biol. Sci.* 373:20160481. 10.1098/rstb.2016.0481 29158311 PMC5717436

[B18] Doyle L. M. Wang M. Z. (2019). Overview of extracellular vesicles, their origin, composition, purpose, and methods for exosome isolation and analysis. *Cells* 8:727. 10.3390/cells8070727 31311206 PMC6678302

[B19] Emambux S. Tachon G. Junca A. Tougeron D. (2018). Results and challenges of immune checkpoint inhibitors in colorectal cancer. *Cell* 18 561–573. 10.1080/14712598.2018.1445222 29471676

[B20] Emig D. Salomonis N. Baumbach J. Lengauer T. Conklin B. R. Albrecht M. (2010). AltAnalyze and domaingraph: analyzing and visualizing exon expression data. *Nucleic Acids Res.* 38 W755–W762. 10.1093/nar/gkq405 20513647 PMC2896198

[B21] Enjeti A. K. Ariyarajah A. D’Crus A. Seldon M. Lincz L. F. (2017). Circulating microvesicle number, function and small RNA content vary with age, gender, smoking status, lipid and hormone profiles. *Thromb. Res.* 156 65–72. 10.1016/j.thromres.2017.04.019 28600979

[B22] Fan J. Wei Q. Koay E. J. Liu Y. Ning B. Bernard P. W. (2018). Chemoresistance transmission via exosome-mediated EphA2 transfer in pancreatic cancer. *Theranostics* 8 5986–5994. 10.7150/thno.26650 30613276 PMC6299429

[B23] Hanyuda A. Ogino S. Qian Z. R. Nishihara R. Song M. Mima K. (2016). Body mass index and risk of colorectal cancer according to tumor lymphocytic infiltrate. *Intern. J. Cancer* 139 854–868. 10.1002/ijc.30122 27037951 PMC5328655

[B24] Herter S. Morra L. Schlenker R. Sulcova J. Fahrni L. Waldhauer I. (2017). A novel three-dimensional heterotypic spheroid model for the assessment of the activity of cancer immunotherapy agents. *Cancer Immunol. Immunother.* 66, 129–140. 10.1007/s00262-016-1927-1 27858101 PMC5222939

[B25] Hon K. W. Ab-Mutalib N. S. Abdullah N. M. A. Jamal R. Abu N. (2019). Extracellular Vesicle-derived circular RNAs confers chemoresistance in colorectal cancer. *Sci. Rep.* 9:16497. 10.1038/s41598-019-53063-y 31712601 PMC6848089

[B26] Ilavská S. Horváthová M. Szabová M. Nemessányi T. Jahnová E. Tulinská J. (2012). Association between the human immune response and body mass index. *Hum. Immunol.* 73 480–485. 10.1016/j.humimm.2012.02.023 22426256

[B27] Ji J. Yin Y. Ju H. Xu X. Liu W. Fu Q. (2018). Long non-coding RNA Lnc-Tim3 exacerbates CD8 T cell exhaustion via binding to Tim-3 and inducing nuclear translocation of Bat3 in HCC. *Cell Death Dis.* 9 478–478. 10.1038/s41419-018-0528-7 29706626 PMC5924754

[B28] Jochem C. Leitzmann M. (2016). Obesity and colorectal cancer. recent results in cancer research. *Fortschritte Krebsforsch. Prog. Recherch. Cancer* 208 17–41. 10.1007/978-3-319-42542-9_2 27909900

[B29] Kalia V. Sarkar S. (2018). Regulation of effector and memory CD8 T cell differentiation by IL-2—A balancing act. *Front. Immunol.* 9:2987. 10.3389/fimmu.2018.02987 30619342 PMC6306427

[B30] Kanehisa M. Furumichi M. Tanabe M. Sato Y. Morishima K. (2016). KEGG: new perspectives on genomes, pathways, diseases and drugs. *Nucleic Acids Res.* 45 D353–D361. 10.1093/nar/gkw1092 27899662 PMC5210567

[B31] Kanehisa M. Sato Y. Furumichi M. Morishima K. Tanabe M. (2019). New approach for understanding genome variations in KEGG. *Nucleic Acids Res.* 47 D590–D595. 10.1093/nar/gky962 30321428 PMC6324070

[B32] Kim A. Shah A. S. Nakamura T. (2018). Extracellular vesicles: a potential novel regulator of obesity and its associated complications. *Children* 5:152. 10.3390/children5110152 30445758 PMC6262587

[B33] Kotzin J. J. Iseka F. Wright J. Basavappa M. G. Clark M. L. Ali M.-A. (2019). The long noncoding RNA Morrbid regulates CD8 T cells in response to viral infection. *Proc. Natl. Acad. Sci. U.S.A.* 116 11916–11925. 10.1073/pnas.1819457116 31138702 PMC6575676

[B34] Liao Y. Wang J. Jaehnig E. J. Shi Z. Zhang B. (2019). WebGestalt 2019: gene set analysis toolkit with revamped UIs and APIs. *Nucleic Acids Res.* 47 W199–W205. 10.1093/nar/gkz401 31114916 PMC6602449

[B35] Lim J. U. Li J. H. Kim J. S. Hwang Y. I. Kim T. Lim S. Y. (2017). Comparison of World Health Organization and Asia-Pacific body mass index classifications in COPD patients. *Int. J. Chron. Obstruct. Pulmon. Dis.* 12 2465–2475. 10.2147/COPD.S141295 28860741 PMC5571887

[B36] Ludwig S. Floros T. Theodoraki M. N. Hong C. S. Jackson E. K. Lang S. (2017). Suppression of lymphocyte functions by plasma exosomes correlates with disease activity in patients with head and neck cancer. *Clin. Cancer Res.* 23 4843–4854. 10.1158/1078-0432.CCR-16-2819 28400428 PMC5559308

[B37] Maimela N. R. Liu S. Zhang Y. (2019). Fates of CD8+ T cells in tumor microenvironment. *Comput. Struct. Biotechnol. J.* 17 1–13. 10.1016/j.csbj.2018.11.004 30581539 PMC6297055

[B38] Mallard E. Vernel-Pauillac F. Velu T. Lehmann F. Abastado J.-P. Salcedo M. (2004). IL-2 production by virus- and tumor-specific human CD8 T cells is determined by their fine specificity. *J. Immunol.* 172 3963–3970. 10.4049/jimmunol.172.6.3963 15004205

[B39] Martinez-Useros J. Garcia-Foncillas J. (2016). Obesity and colorectal cancer: molecular features of adipose tissue. *J. Transl. Med.* 14:21. 10.1186/s12967-016-0772-5 26801617 PMC4722674

[B40] Maybruck B. T. Pfannenstiel L. W. Diaz-Montero M. Gastman B. R. (2017). Tumor-derived exosomes induce CD8+ T cell suppressors. *J. Immunother. Cancer* 5:65. 10.1186/s40425-017-0269-7 28806909 PMC5556362

[B41] Meyerhardt J. A. Catalano P. J. Haller D. G. Mayer R. J. Benson A. B. III Macdonald J. S. (2003). Influence of body mass index on outcomes and treatment-related toxicity in patients with colon carcinoma. *Cancer* 98 484–495. 10.1002/cncr.11544 12879464

[B42] Mojic M. Takeda K. Hayakawa Y. (2017). The dark side of IFN-γ: its role in promoting cancer immunoevasion. *Int. J. Mol. Sci.* 19:89. 10.3390/ijms19010089 29283429 PMC5796039

[B43] Muller L. Mitsuhashi M. Simms P. Gooding W. E. Whiteside T. L. (2016). Tumor-derived exosomes regulate expression of immune function-related genes in human T cell subsets. *Sci. Rep.* 6:20254. 10.1038/srep20254 26842680 PMC4740743

[B44] Muller L. Simms P. Hong C.-S. Nishimura M. I. Jackson E. K. Watkins S. C. (2017). Human tumor-derived exosomes (TEX) regulate Treg functions via cell surface signaling rather than uptake mechanisms. *Oncoimmunology* 6:e1261243. 10.1080/2162402X.2016.1261243 28919985 PMC5593709

[B45] Murphy T. K. Calle E. E. Rodriguez C. Kahn H. S. Thun M. J. (2000). Body mass index and colon cancer mortality in a large prospective study. *Am. J. Epidemiol.* 152 847–854. 10.1093/aje/152.9.847 11085396

[B46] Newman A. M. Steen C. B. Liu C. L. Gentles A. J. Chaudhuri A. A. Scherer F. (2019). Determining cell type abundance and expression from bulk tissues with digital cytometry. *Nat. Biotechnol.* 37 773–782. 10.1038/s41587-019-0114-2 31061481 PMC6610714

[B47] Ni L. Lu J. (2018). Interferon gamma in cancer immunotherapy. *Cancer Med.* 7 4509–4516. 10.1002/cam4.1700 30039553 PMC6143921

[B48] Othman N. Jamal R. Abu N. (2019). Cancer-derived exosomes as effectors of key inflammation-related players. *Front. Immunol.* 10:2103. 10.3389/fimmu.2019.02103 31555295 PMC6737008

[B49] Park Y. Peterson L. L. Colditz G. A. (2018). The plausibility of obesity paradox in cancer-point. *Cancer Res.* 78 1898–1903. 10.1158/0008-5472.CAN-17-3043 29654151 PMC5903573

[B50] Picard E. Verschoor C. P. Ma G. W. Pawelec G. (2020). Relationships between immune landscapes, genetic subtypes and responses to immunotherapy in colorectal cancer. *Front. Immunol.* 11:369. 10.3389/fimmu.2020.00369 32210966 PMC7068608

[B51] Ragni E. Banfi F. Barilani M. Cherubini A. Parazzi V. Larghi P. (2017). Extracellular vesicle-shuttled mRNA in mesenchymal stem cell communication. *Stem Cells* 35 1093–1105. 10.1002/stem.2557 28164431

[B52] Riihimäki M. Hemminki A. Sundquist J. Hemminki K. (2016). Patterns of metastasis in colon and rectal cancer. *Sci. Rep.* 6:29765. 10.1038/srep29765 27416752 PMC4945942

[B53] Schmidt F. M. Weschenfelder J. Sander C. Minkwitz J. Thormann J. Chittka T. (2015). Inflammatory cytokines in general and central obesity and modulating effects of physical activity. *PLoS One* 10:e0121971. 10.1371/journal.pone.0121971 25781614 PMC4363366

[B54] Shahjehan F. Merchea A. Cochuyt J. J. Li Z. Colibaseanu D. T. Kasi P. M. (2018). Body mass index and long-term outcomes in patients with colorectal cancer. *Front. Oncol.* 8:620. 10.3389/fimmu.2020.00620 30631753 PMC6315135

[B55] Sharma P. Diergaarde B. Ferrone S. Kirkwood J. M. Whiteside T. L. (2020). Melanoma cell-derived exosomes in plasma of melanoma patients suppress functions of immune effector cells. *Sci. Rep.* 10:92. 10.1038/s41598-019-56542-4 31919420 PMC6952363

[B56] Sinicrope F. A. Foster N. R. Yothers G. Benson A. Seitz J. F. Labianca R. (2013). Body mass index at diagnosis and survival among colon cancer patients enrolled in clinical trials of adjuvant chemotherapy. *Cancer* 119 1528–1536. 10.1002/cncr.27938 23310947 PMC3769640

[B57] Szatanek R. Baj-Krzyworzeka M. Zimoch J. Lekka M. Siedlar M. Baran J. (2017). The methods of choice for extracellular vesicles (EVs) characterization. *Int. J. Mol. Sci.* 18:1153. 10.3390/ijms18061153 28555055 PMC5485977

[B58] Tran C. G. Hill E. E. Jensen B. Stark A. C. Flannery M. Berg D. J. (2018). Survival benefit of obesity in stage IV colorectal cancer: better tolerability of chemotherapy? *J. Clin. Oncol.* 36:e15629 10.1200/JCO.2018.36.15_suppl.e15629

[B59] Turbitt W. J. Buchta Rosean C. Weber K. S. Norian L. A. (2020). Obesity and CD8 T cell metabolism: implications for anti-tumor immunity and cancer immunotherapy outcomes. *Immunol. Rev.* 295 203–219. 10.1111/imr.12849 32157710 PMC7416819

[B60] Ujvari B. Jacqueline C. Misse D. Amar V. Fitzpatrick J. C. Jennings G. (2019). Obesity paradox in cancer: is bigger really better? *Evol. Appl.* 12 1092–1095. 10.1111/eva.12790 31293625 PMC6597865

[B61] Volpe E. Sambucci M. Battistini L. Borsellino G. (2016). Fas–Fas Ligand: checkpoint of T cell functions in multiple sclerosis. *Front. Immunol.* 7:382. 10.3389/fimmu.2016.00382 27729910 PMC5037862

[B62] Wang Z. Aguilar E. G. Luna J. I. Dunai C. (2019). Paradoxical effects of obesity on T cell function during tumor progression and PD-1 checkpoint blockade. *Nat. Med.* 25 141–151. 10.1038/s41591-018-0221-5 30420753 PMC6324991

[B63] Wen S. W. Sceneay J. Lima L. G. Wong C. S. F. Becker M. Krumeich S. (2016). The biodistribution and immune suppressive effects of breast cancer–derived exosomes. *Cancer Res.* 76:6816. 10.1158/0008-5472.CAN-16-0868 27760789

[B64] Whiteside T. L. (2017). Exosomes carrying immunoinhibitory proteins and their role in cancer. *Clin. Exper. Immunol.* 189 259–267. 10.1111/cei.12974 28369805 PMC5543496

[B65] WHO Expert Consultation (2004). Appropriate body-mass index for Asian populations and its implications for policy and intervention strategies. *Lancet* 363 157–163. 10.1016/S0140-6736(03)15268-3 14726171

[B66] Zhou Y. Zhu Y. Xie Y. Ma X. (2019). The role of long non-coding RNAs in immunotherapy resistance. *Front. Oncol.* 9:1292. 10.3389/fonc.2019.01292 31850199 PMC6892777

